# Supplementary material for: Graft survival of major histocompatibility complex deficient stem cell-derived retinal cells
Source: Commun Med (Lond). 2024 Sep 30;4:187. doi: 10.1038/s43856-024-00617-5 (PMC11442691; doi:10.1038/s43856-024-00617-5)
Supplement: Supplementary file 2 — Supplementary information [file 43856_2024_617_MOESM2_ESM.pdf]

Supplementary information

Graft survival of major histocompatibility complex deficient stem cell-derived retinal cells

Masaaki Ishida[1,2], Tomohiro Masuda[1], Noriko Sakai[1], Yoko Nakai-Futatsugi[1,3**], Hiroyuki Kamao[4], Takashi Shiina[5], Masayo Takahashi[1,3,6], Sunao Sugita[1,3,6*]

Supplementary Fig. 1-12

Supplementary Table 1-2

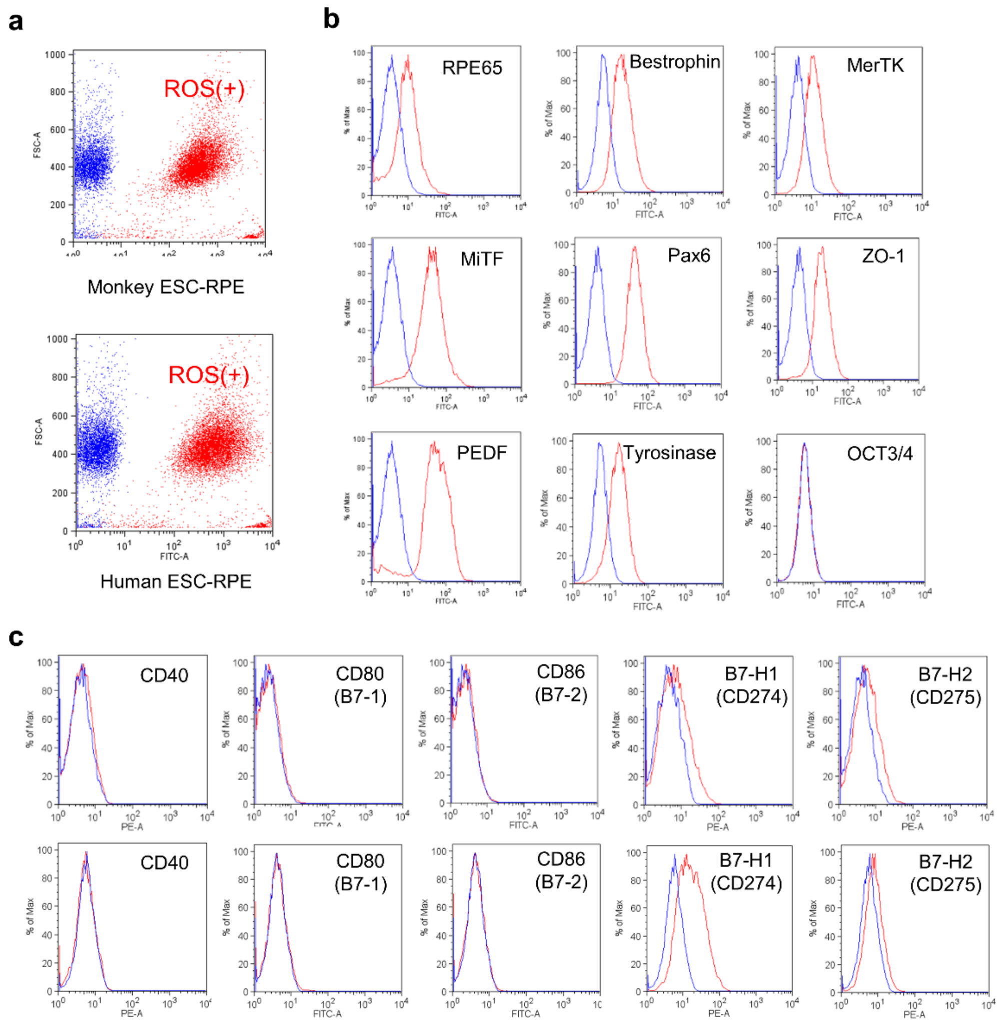

Supplementary Fig. 1 Characterization of CMK6 ESC-RPE cells.

(a) Phagocytosis of monkey CMK6-derived ESC-RPE and normal human-derived ESC-RPE. Both ESC-RPE cells were cultured with FITC-labeled porcine shed photoreceptor rod outer segments (ROS) at 37°C. They were analyzed using flow cytometry after an incubation (24hr). Both monkey and human ESC-RPE cells had normal function of phagocytosis. (**b**) Expression of RPE-specific markers of monkey CMK6 ESC-RPE (mESC-RPE). mESC-RPE cells were stained with anti-RPE65, MiTF, PEDF, bestrophin, Pax6, tyrosinase, MerTK, ZO-1, and OCT3/4 antibodies (red). Isotype control is shown in blue. (**c**) Expression of co-stimulatory molecules on monkey CMK6 (upper) and human (lower) ESC-RPE. Both ESC-RPE cells were incubated with recombinant IFN-γ (100 ng/mL) for 48 hr. There was no obvious difference between monkey CMK6 and normal human mESC-RPE cells.

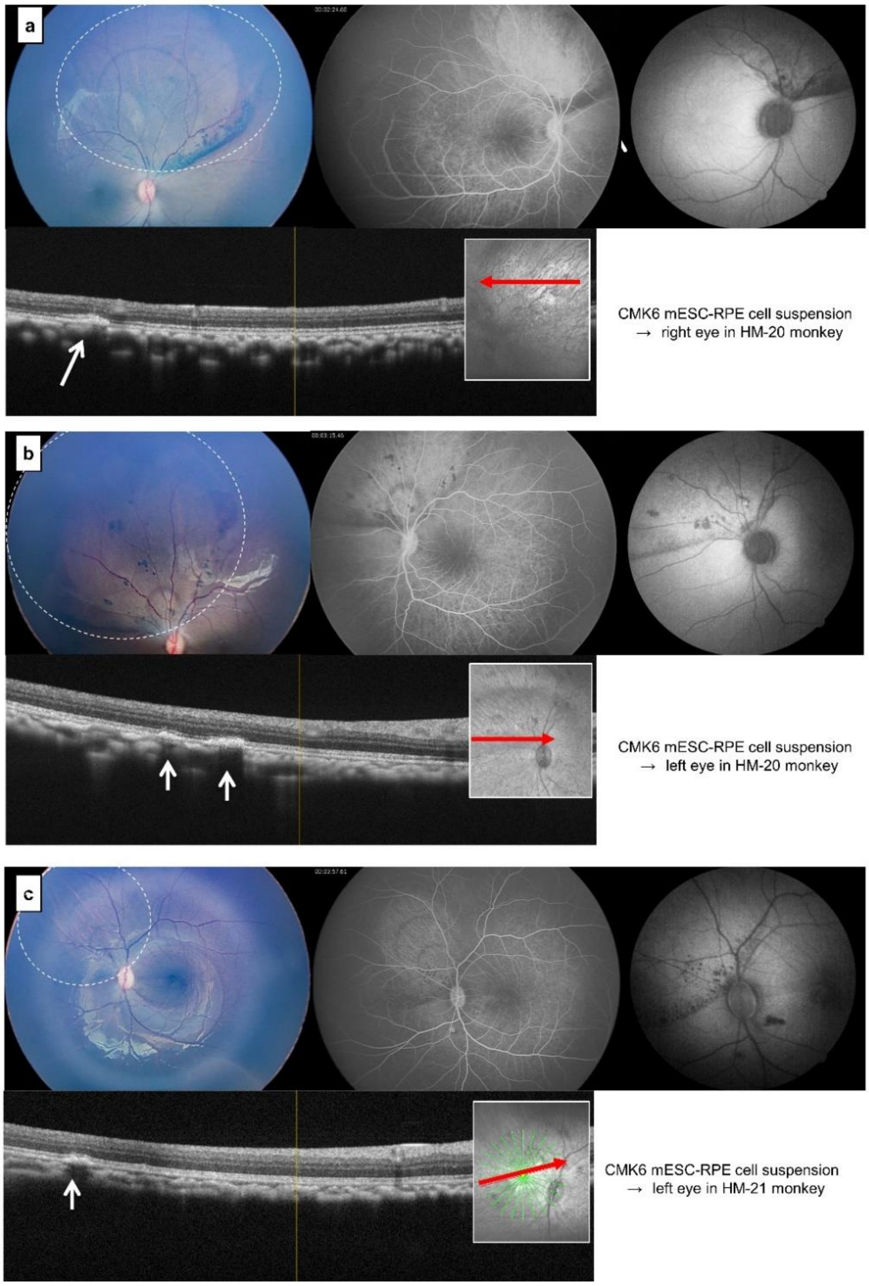

Supplementary Fig. 2 Transplantation of CMK6 ESC-RPE cell suspension allografts.

(**a-c**) CMK6-derived mESC-RPE cell suspension was transplanted into the subretinal space of monkey HM-20 (a: right eye, b: left eye) and HM-21(c: left eye). Dashed line circle in color picture (left panels) indicates retinal bleb made for injection. Shown are fluorescein angiography (middle), fundus auto-fluorescent (right) and OCT (lower) at 6 months after transplantation. White arrows: mESC-RPE grafts. Many sheet-like grafted RPE cells were observed by OCT, while no signs of rejection were found.

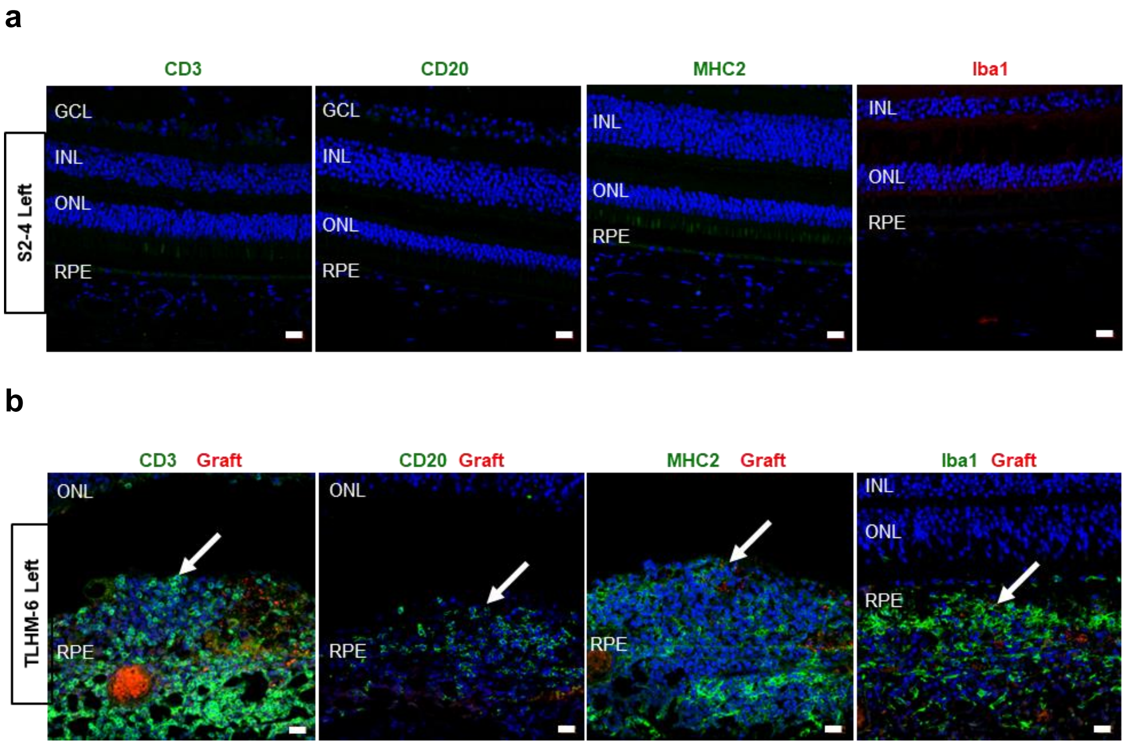

Supplementary Fig. 3 Immune response of normal retina and retina with extreme rejection.

Normal retina without transplantation[27] (**a**) and retina that showed extreme immune rejection after allogenic RPE transplantation[31] (**b**) were stained with CD3 (T-cells), CD20 (B-cells), MHCII (antigen presenting cells) and Iba1 (microgia/macrophages). Obvious invasion of these inflammatory cells into the RPE layer accompanied the rejection (**b**), while only a few Iba1^{+} cells were detected in normal retia (**a**). Scale bars: 20 μm.

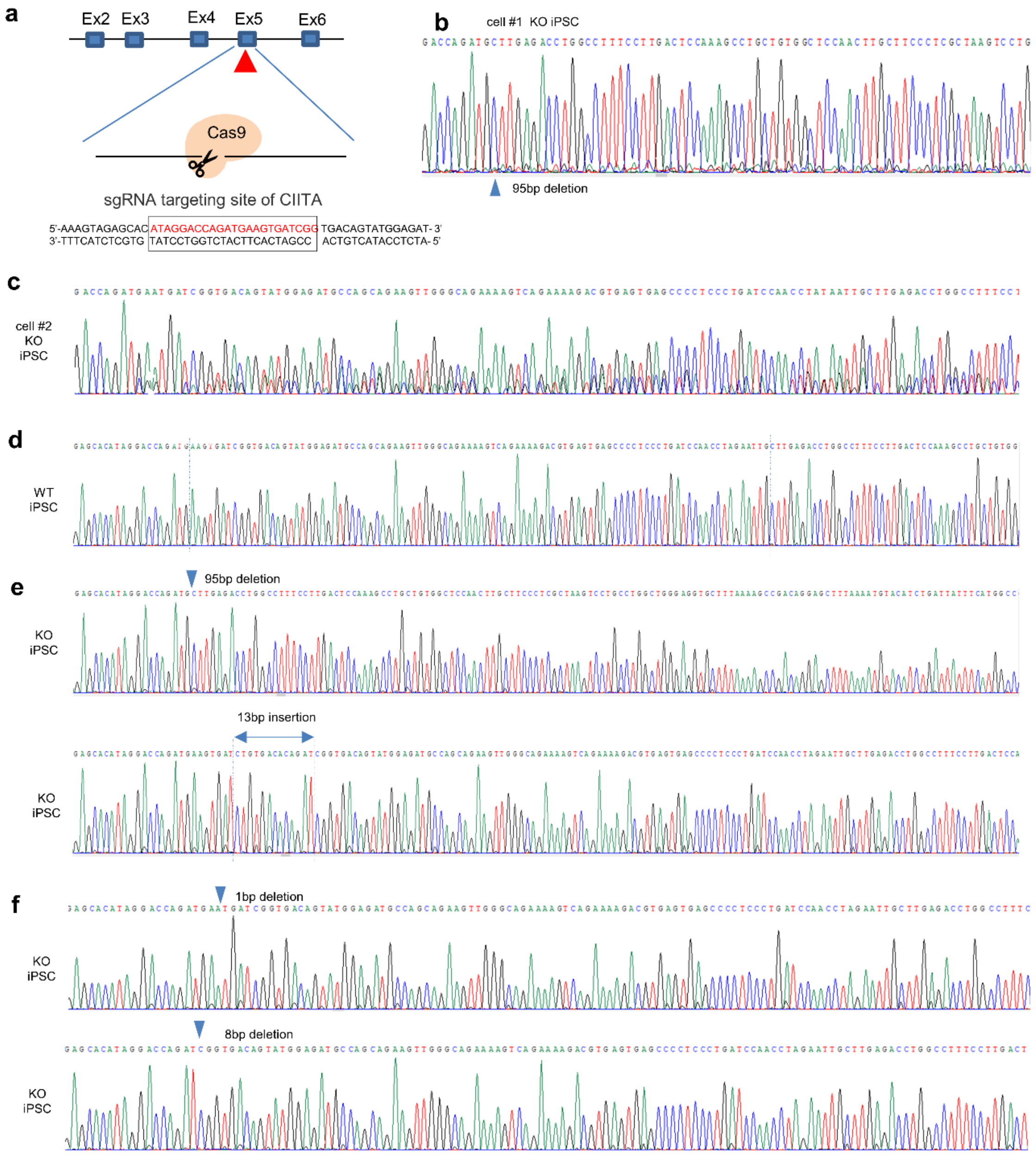

Supplementary Fig. 4 Generation of *CIITA* knockout monkey iPSCs and RPE cells.

(a) CRISPR - Cas9 system designed for *CIITA* gene knockout. Red arrow head indicates the guide RNA (sgRNA) targeting site of *CIITA* gene.

(b, c) The sequences of *CIITA* knockout monkey iPSCs (cell #1(b) and cell #2(c)).

(d) The sequence of wildtype monkey iPSCs (WT-iPSC). (e, f) The results of TOPO TA cloning of cell #1 (b) and cell #2 (c). The two had different sequences, and neither possessed the sequence of wildtype.

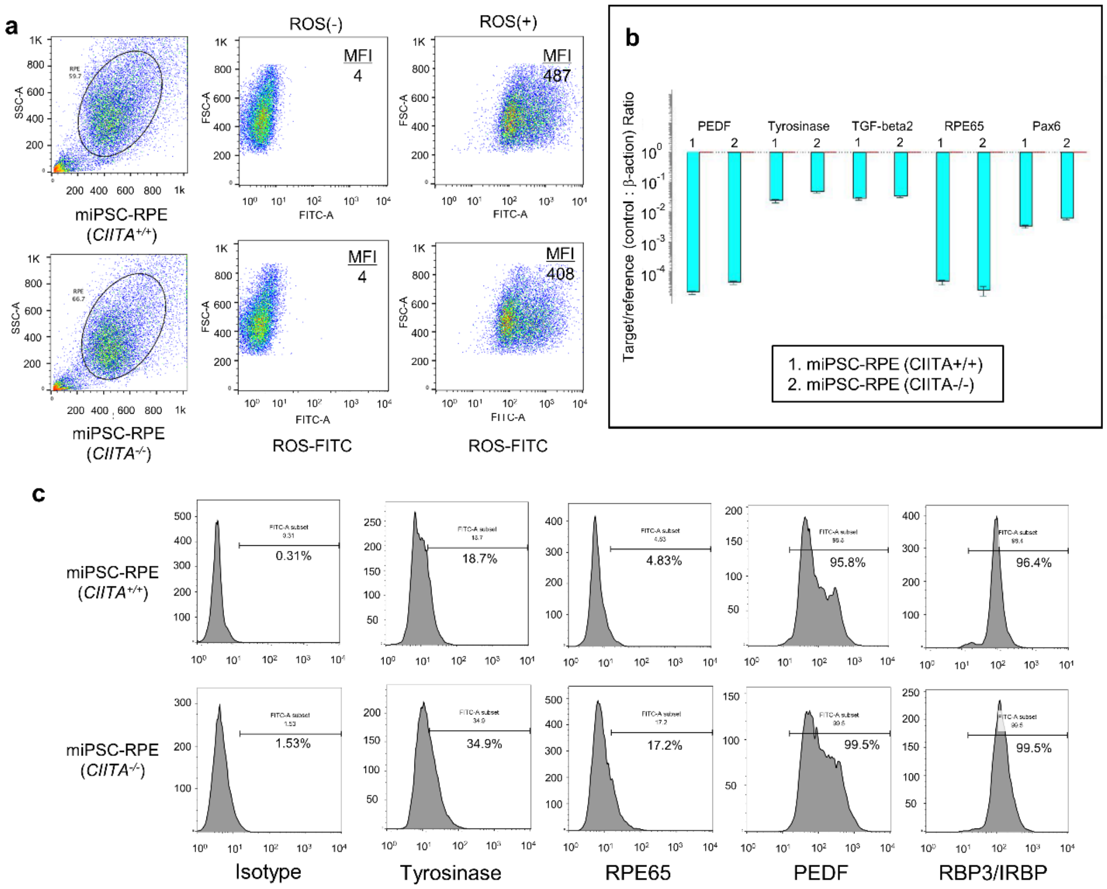

Supplementary Fig. 5 The characteristics of *CIITA*$^{+/+}$ and *CIITA*$^{-/-}$ monkey iPSC-RPE cells.

(**a**) Phagocytosis of *CIITA*$^{+/+}$ and *CIITA*$^{-/-}$ miPSC-RPE cells. Both cells had normal function of phagocytosis. (b) The expressions of RPE-specific markers such as PEDF, Tyrosinase, TGF-b, RPE65 and Pax6 in iPSC-RPE cells were examined by qRT−PCR. There were no statistical differences between *CIITA*$^{+/+}$ and *CIITA*$^{-/-}$ RPE cells (n=3 replicates; Welch’s t-test). Bar represents mean with standard deviation. (c) Expressions of RPE-specific markers in *CIITA*$^{+/+}$ and *CIITA*$^{-/-}$ miPSC-RPE. Both miPSC-RPE cells were stained with anti-tyrosinase, RPE65, PEDF and RBP3/IRBP antibodies.

The FSC/SSC gates were determined by our preliminary study to include the population positive for RPE markers such as RPE65.

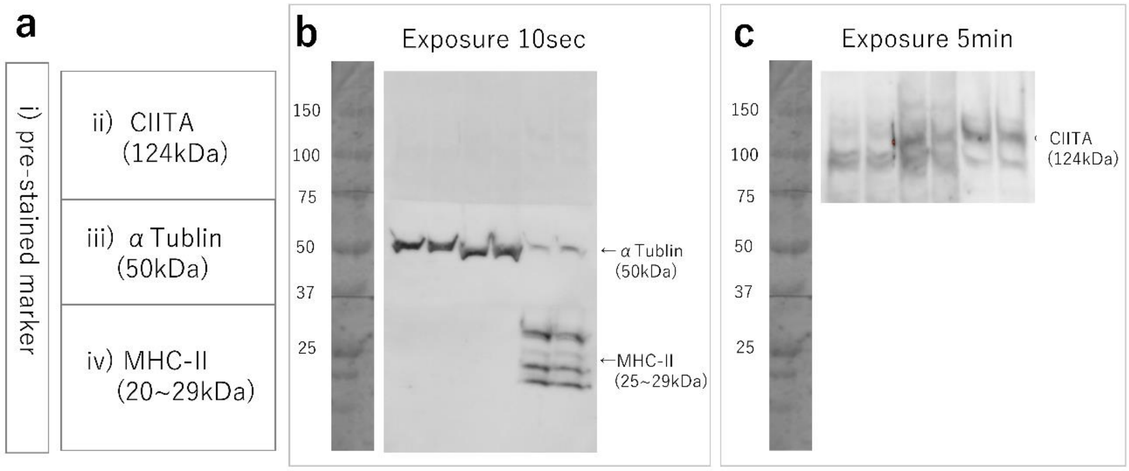

Supplementary Fig. 6 The Full length bolts for the Western blot show in Fig. 3d. (**a**) The blotted membrane was cut in four pieces before the incubation with primary antibodies: i) the first lane blotted with a pre-stained marker (Bio-rad, 161-0373) was cut; ii) was cut just below 75kDa, and incubated with anti-CIITA antibody (GENETEX, GTX129022 1:100); iii) was cut just below 37kDa, and incubated with anti-αTubulin antibody (SIGMA-ALDRICH, DM1A, 1:5000); iv) the remaining was incubated with anti-MHC-II antibody (abeomics, 36-1236 1:500). (**b**) i) shown in (*a*) was imaged with *Digitization/Epi-illumination mode* (LAS 600, GE health care); ii)~iv) shown in (*a*) was imaged with *Chemiluminescence mode* (LAS 600, GE health care) with exposure time of 10 sec. (**c**) ii) shown in (*a*) was imaged with *Chemiluminescence mode* (LAS 600, GE health care) with exposure time of 5 min.

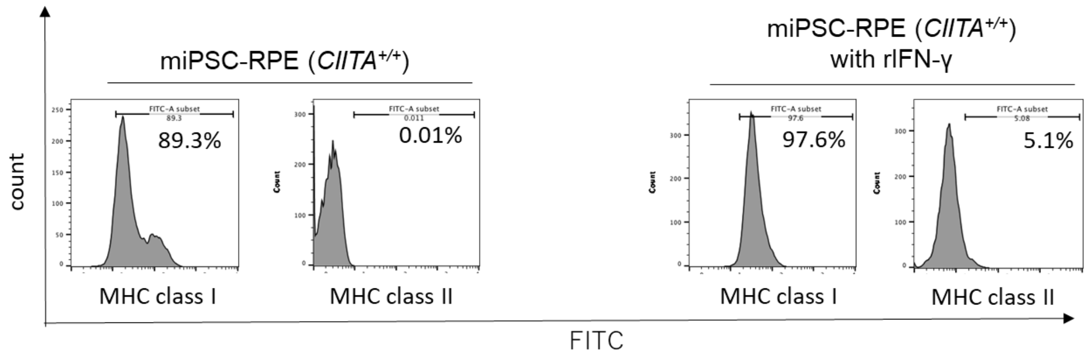

Supplementary Fig. 7 Wildtype *CIITA*$^{+/+}$ miPSC-RPE cells express MHC-II in response to IFN-γ Expressions of MHC-I and -II on the cell surface of *CIITA*$^{+/+}$ miPSC-RPE cells were examined by flowcytometry. Both MHC-I and -II were expressed in response to IFN-γ. This was in contrast to *CIITA*$^{-/-}$ miPSC-RPE cells that lacked the expression of MHC-II as shown in **Fig. 3e**.

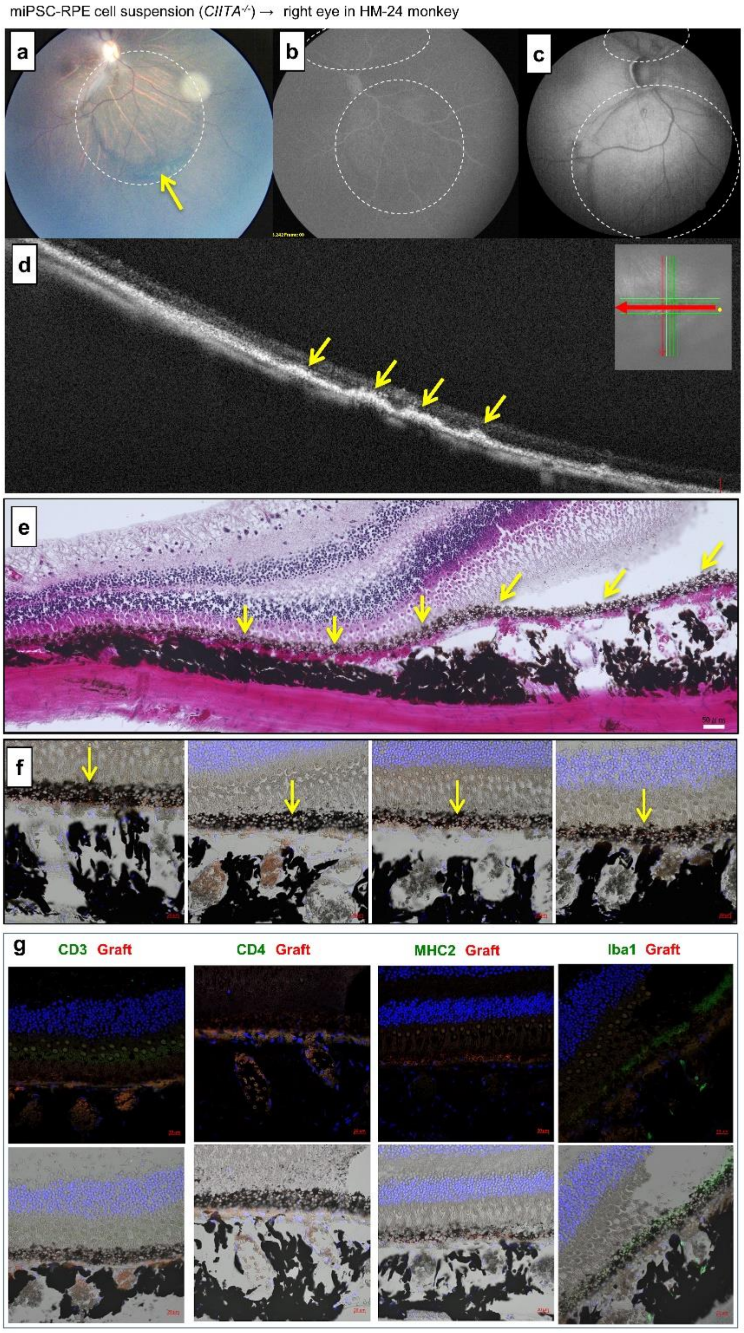

Supplementary Fig. 8 Additional transplantation of *CIITA*$^{-/-}$ monkey iPSC-RPE cells in *in vivo* animal model (HM-24)

Ophthalmologic and histological analysis detected the survival of grafted *CIITA*$^{-/-}$ miPSC-RPE cells in MHC-mismatched transplantation. (a-e) HM-24 was monitored by color fundus pictures (a), fluorescein angiography (b), fundus autofluorescence (c), and optical coherence tomography (d) for 6 months after transplantation. There was no sign of rejection during this observation period. The grafts survived in the subretinal space (yellow arrows). (e) The evaluation of H&E staining at 6 months. Survival of the graft was observed throughout the retinal sections. Scale bar: 50 μm. (f,g) The evaluation of the RPE grafts and immune cells by IHC staining. (f) Graft cells (yellow arrows) in the sections (DAPI staining only; bright field). (g) Inflammatory immune cells such as CD3, CD4, and MHC-II positive cells were not detected in the sections. A few Iba1+ cells were seen around the grafted space (choroid) but grafted RPE cells (red) survived. Scale bars: 20 μm.

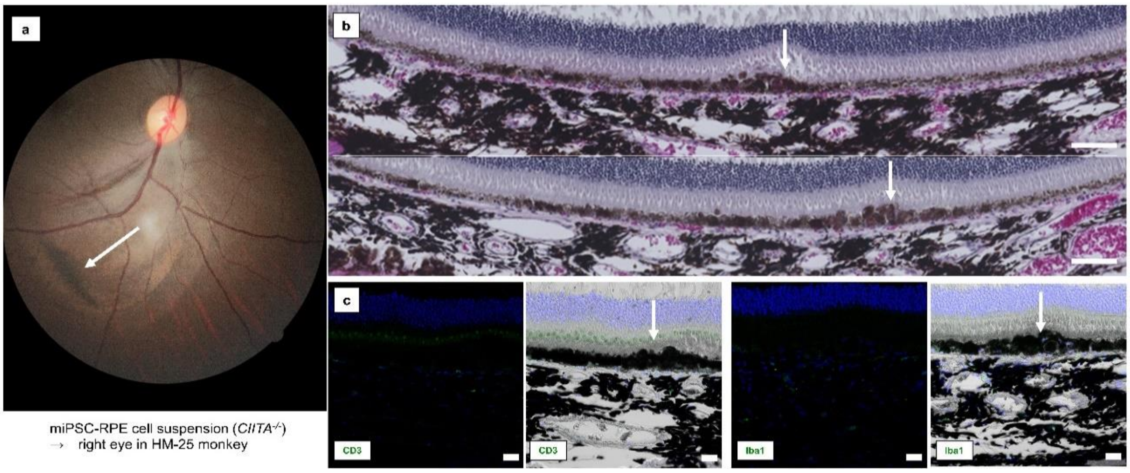

Supplementary Fig. 9 Additional transplantation of *CIITA$^{-/-}$* monkey iPSC-RPE cells in *in vivo* animal model with ROCK inhibitor (HM-25)

Allotransplantation with Rho-associated protein kinase (ROCK) inhibitor. Ophthalmologic and histological analysis detected survival of the graft in another MHC-mismatched transplantation (HM-25). (a) The eyes of HM-25 were monitored by color fundus pictures (a), fluorescein angiography, fundus autofluorescence, and optical coherence tomography (not shown) for 6 months after transplantation. The grafts survived in the subretinal space (white arrows) and there was no sign of immune rejection. (b) The evaluation of H&E staining at 6 months. Survival of the graft was observed throughout the retinal sections. Scale bar: 50 μm. (c) The evaluation of the RPE grafts and immune cells by IHC staining. Graft cells were detected (white arrows) while Inflammatory immune cells such as CD3+ and Iba1+ cells were not detected in the sections. Scale bars: 20 μm.

a

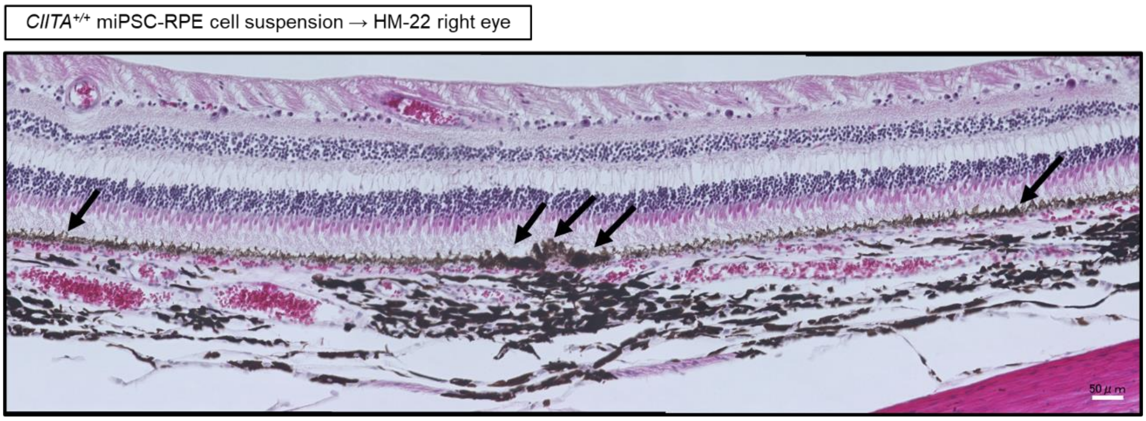

b

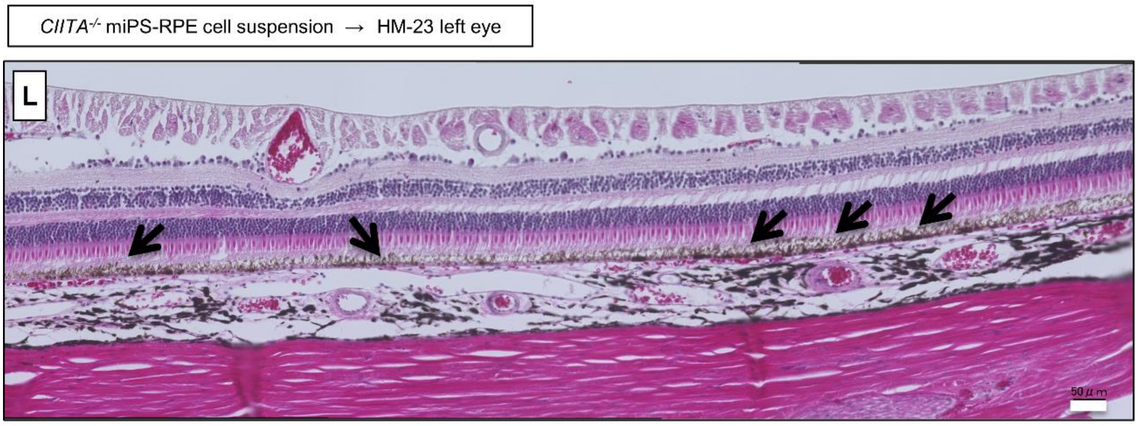

Supplementary Fig. 10 H&E staining of monkey HM-22 and HM-23 transplanted with *CIITA*$^{+/+}$ and *CIITA*$^{-/-}$ miPSC-RPE cells, respectively. Grafted RPE cells were detected by H&E staining in monkey HM-22 (a) and HM-23 (b) 6 month after transplantation of *CIITA*$^{+/+}$ (a) or *CIITA*$^{-/-}$ (b) miPSC-RPE allografts, respectively. Although by either transplantation the grafted RPE cells formed monolayer sheets in the subretinal space (arrows), *CIITA*$^{+/+}$ RPE injection resulted to some extent in less engraftment (a) compared to *CIITA*$^{-/-}$ RPE injection (b). Scale bars: 50 μm.

Transplant	Monkey ID	IHC	
		Iba1 (count)	CD3 (count)
CIITA$^{+/+}$ wildtype	HM-22	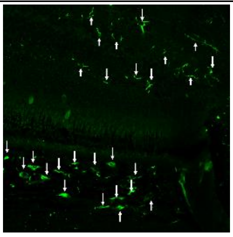 (27)	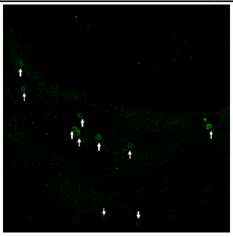 (10)
	K177	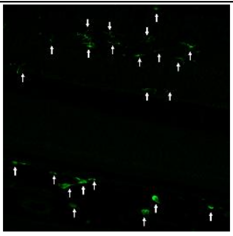 (23)	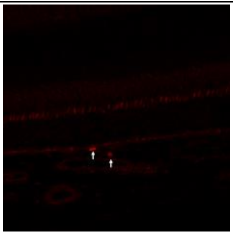 (2)
	S2-4	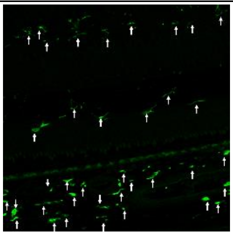 (37)	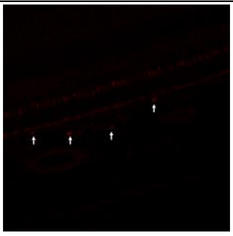 (4)
CIITA$^{-/-}$ MHCII-KO	HM-23	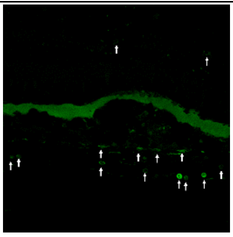 (14)	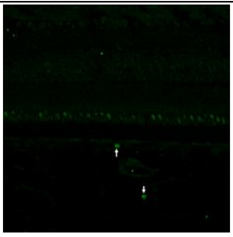 (2)
	HM-24	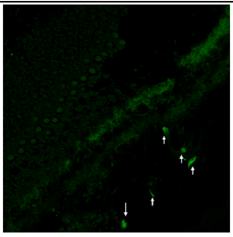 (5)	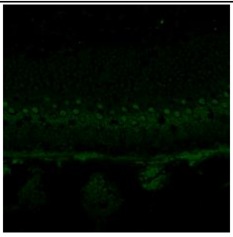 (0)
	HM-25	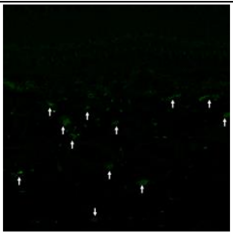 (12)	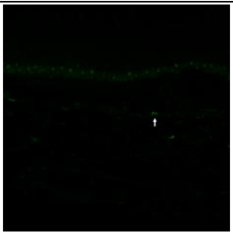 (1)

Supplementary Fig. 11 IHC images used for quantification Inflammatory cells invaded into the grafted layer was quantified in monkeys that received *CIITA*$^{+/+}$ wildtype (n=3 monkeys) and *CIITA*$^{-/-}$ MHCII-KO (n=3 monkeys) transplantation. Results are in **Fig 5c**.

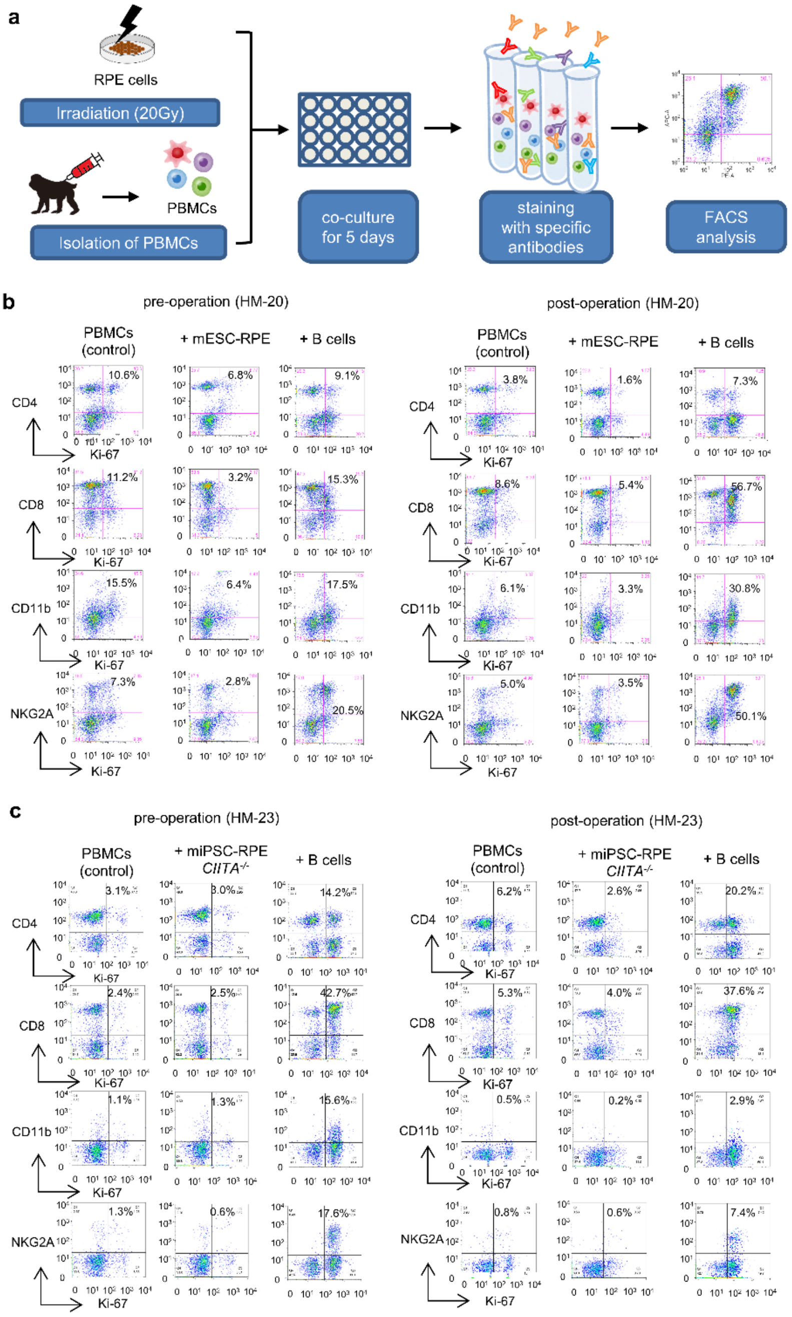

Supplementary Fig. 12 *In vitro* immunological blood tests after *CIITA*$^{+/+}$ or *CIITA*$^{-/-}$ miPSC-RPE cell transplantation

(**a**) The schema of lymphocytes-graft cell immune reaction (LGIR) test with grafted RPE cells and recipient blood lymphocytes from the monkeys. PBMC in the monkey were collected before and 8~12 weeks after operation, and were cultured with graft RPE cells, as well as B95-8 B cells (allogeneic monkey cells) as a positive control (PC). After 5 days of co-culture, the PBMC samples were analyzed using a flow cytometer. (**b,c**) The results of LGIR test for HM-20, a monkey transplanted with CMK6-derived mESC-RPE (b) and HM-23, a monkey transplanted with *CIITA*$^{-/-}$ miPSC-RPE (c). In both cases, there was no proliferation of immune cells (CD4^{+}, CD8^{+}, CD11b^{+}, and NKG2A^{+} cells) by co-culture with transplanted RPE cells, whereas highly proliferative immune cells were detected by co-culture with PC cells. Numbers (%) indicate double-positive cells. The FSC/SSC gates were determined by our preliminary study to include the population positive for lymphocyte markers (e.g. CD4, CD8, CD11b or NKG2A). For Ki-67 staining, the gate was extended to higher FSC/SSC to include proliferating cells.

Supplementary Table 1 Guide RNA sequences and primer sequences

RFXANK (NM_003721)	**grid ID**	**gRNA seq**
exon3	hs058912694	TGAGCCTGTGAATCCTGAACCGG
exon3	hs058912702	GCATCCGGTTCAGGATTCACAGG
exon4	hs058912772	ACTCCACCACTCTCACCAACCGG
exon4	hs058912782	ACACCTCGTTCCCTCGCTGCCGG
exon7	hs058912966	GCCTGTGCTGGCCAGCGACAGGG
exon6	hs058912898	TGAGACCGTTCGCTTCCTGCTGG

CIITA (NM_000246)	**grid ID**	**gRNA seq**
exon2	hs044825285	GGTCCATCTGGTCATAGAAGTGG
exon2	hs044825273	TCCAGGTAGCCACCTTCTAGGGG
exon3	hs044825354	GCTGAACTGGTCGCAGTTGATGG
exon3	hs044825358	GGAAGGTGATGAAGAGACCAGGG
exon3	hs044825350	TCAACTGCGACCAGTTCAGCAGG
exon5	hs044825594	ATAGGACCAGATGAAGTGATCGG

gRNA	forward		reverse	
#1 (hs044825548)	#1	GAAGCCCAGAGTGGTTAAGTAG	#1	GGGTCTGACTGCCCATAAAT
	#2	GTAGGGCTAGTGGAGAGTAGAG	#2	CCCAAGGAAGAAGTCAGGAAA
#2 (hs044825594)	#3	CCTTGTTGATTGACTGCACTTT	#3	TTCTGCTGGCATCTCCATAC
	#4	GCCTGCATTTCCTGACTTCT	#4	AGGCCTTGTGGCATGTATTT
#3 (hs058912772)	#5	CTGAGGCAGGTGGATGATTT	#5	AGGTCACACAGACAGGTAGATA
	#6	CGCCTATAATCCCAGCTACTTG	#6	CCTCCTACTCCTCTCCTTGTT
#4 (hs058912898)	#7	CCACGTGTGTGCTTGTATCTA	#7	TCCCAAAGTGCTGGGATTAC
	#8	TATCTACCTGTCTGTGTGACCT	#8	CCTCCAGAGTACCTGGGATTA

Supplementary Table 2 Materials for IHC and Flow cytometry

antibody	species	dilution	catalog	source
Anti-human HLA-class I, FITC	Mouse	10 μl/test	F5662	Sigma-Aldrich
Anti-human HLA-class II, FITC	Mouse	10 μl/test	MCA2497F	AbD Serotec
Anti-human CD40, PE	Mouse	5 μl/test	313006	BioLegend
Anti-human CD80 (B7-1), PE	Mouse	5 μl/test	11-0809	eBioscience
Anti-human CD86 (B7-2), Alexa Fluor 488	Mouse	5 μl/test	53-0869	eBioscience
Anti-bestrophin	Mouse	10 μl/test	MAB5466	Millipore
Anti-MerTK	rabbit	10 μg/test	sc-67280	Santa Cruz
Anti-MiTF	Mouse	10 μg/test	ab80651	Abcam
Anti-Oct3/4	Mouse	x200	sc-5279	Santa Cruz
Anti-Pax6	rabbit	x200	PRB-278P	Covance
Anti-PEDF	Mouse	10 μg/test	ab115489	Abcam
Anti-RPE65	Mouse	10 μg/test	MAB5428	Millipore
Anti-tyrosinase	Mouse	x200	ab738	Abcam
Anti-ZO-1	rabbit	x100	61-7300	Thermo Fisher
Mouse IgG1, isotype control	-	1 μg/test	ab170190	Abcam
Mouse IgG1, isotype control, PE	-	5 μl/test	400112	BioLegend
Mouse IgG2a, isotype control, FITC	-	2 μl/test	400210	BioLegend
Mouse IgG2b, isotype control, APC	-	5 μl/test	400322	BioLegend
Rabbit IgG, isotype control	-	x200	02-6102	Thermo Fisher
Anti-human CD4, APC	Mouse	5 μl/test	317416	BioLegend
Anti-human CD8a, APC	Mouse	5 μl/test	17-0088	eBioscience
Anti-human CD11b, APC	Rat	5 μl/test	13-091-241	Miltenyi Biotec
Anti-human CD20, APC	Mouse	5 μl/test	130-097-619	Miltenyi Biotec
Anti-human Ki-67, PE	Mouse	5 μl/test	350504	BioLegend
Anti-CD3	Rabbit	x100	ab16669	Abcam
Anti-Iba1	Rabbit	x1000	019-19741	Wako
Anti-CD4	Rabbit	X100	Ab133616	Abcam
Anti-MHC class II (HLA-DP, DQ, DR)	Mouse	x100	M0775	DakoCytomation
Alexa Fluor 488 anti-rabbit IgG (H+L)	Goat	x1000	A11034	tab
Alexa Fluor 546 anti-rabbit IgG (H+L)	Goat	x1000	A11035	Invitrogen
Alexa Fluor 647 anti-rabbit IgG (H+L)	Goat	x1000	A21245	Invitrogen
Alexa Fluor 546 anti-mouse IgG (H+L)	Goat	x1000	A11030	Invitrogen
