# Supplementary material for: Graft survival of major histocompatibility complex deficient stem cell-derived retinal cells
Source: Commun Med (Lond). 2024 Sep 30;4:187. doi: 10.1038/s43856-024-00617-5 (PMC11442691; doi:10.1038/s43856-024-00617-5)
Supplement: Supplementary file 3 — Description of Additional Supplementary Files [file 43856_2024_617_MOESM3_ESM.pdf]

Description of Additional Supplementary Files

File name: Supplementary Data 1

File description: Results of MHC genotyping

File name: Supplementary Data 2

File description: The numerical results underlying Fig. 5c and Fig. 7c
