# Supplementary material for: Graft survival of major histocompatibility complex deficient stem cell-derived retinal cells
Source: Commun Med (Lond). 2024 Sep 30;4:187. doi: 10.1038/s43856-024-00617-5 (PMC11442691; doi:10.1038/s43856-024-00617-5)
Supplement: Supplementary file 6 — Reporting Summary [file 43856_2024_617_MOESM6_ESM.pdf]

nature portfolio

Corresponding author(s): Sunao Sugita
Yoko Nakai-Futatsugi

Last updated by author(s): Aug 2, 2024

Reporting Summary

Nature Portfolio wishes to improve the reproducibility of the work that we publish. This form provides structure for consistency and transparency in reporting. For further information on Nature Portfolio policies, see our Editorial Policies and the Editorial Policy Checklist.

Statistics

For all statistical analyses, confirm that the following items are present in the figure legend, table legend, main text, or Methods section.

n/a	Confirmed	
☐	☒	The exact sample size (*n*) for each experimental group/condition, given as a discrete number and unit of measurement
☐	☒	A statement on whether measurements were taken from distinct samples or whether the same sample was measured repeatedly
☐	☒	The statistical test(s) used AND whether they are one- or two-sided<br>*Only common tests should be described solely by name; describe more complex techniques in the Methods section.*
☐	☒	A description of all covariates tested
☐	☒	A description of any assumptions or corrections, such as tests of normality and adjustment for multiple comparisons
☐	☒	A full description of the statistical parameters including central tendency (e.g. means) or other basic estimates (e.g. regression coefficient) AND variation (e.g. standard deviation) or associated estimates of uncertainty (e.g. confidence intervals)
☐	☒	For null hypothesis testing, the test statistic (e.g. *F*, *t*, *r*) with confidence intervals, effect sizes, degrees of freedom and *P* value noted<br>*Give P values as exact values whenever suitable.*
☒	☐	For Bayesian analysis, information on the choice of priors and Markov chain Monte Carlo settings
☒	☐	For hierarchical and complex designs, identification of the appropriate level for tests and full reporting of outcomes
☒	☐	Estimates of effect sizes (e.g. Cohen's *d*, Pearson's *r*), indicating how they were calculated

Our web collection on statistics for biologists contains articles on many of the points above.

Software and code

Policy information about availability of computer code

Data collection	BD FACSDiva v6.0; LCS480 1.5.1.62
Data analysis	Microsoft Excel 2019; FlowJo v10

For manuscripts utilizing custom algorithms or software that are central to the research but not yet described in published literature, software must be made available to editors and reviewers. We strongly encourage code deposition in a community repository (e.g. GitHub). See the Nature Portfolio guidelines for submitting code & software for further information.

Data

Policy information about availability of data

All manuscripts must include a data availability statement. This statement should provide the following information, where applicable:

- Accession codes, unique identifiers, or web links for publicly available datasets
- A description of any restrictions on data availability
- For clinical datasets or third party data, please ensure that the statement adheres to our policy

The data obtained in this study are available from the corresponding authors upon reasonable request.

Field-specific reporting

Please select the one below that is the best fit for your research. If you are not sure, read the appropriate sections before making your selection.

☒ Life sciences ☐ Behavioural & social sciences ☐ Ecological, evolutionary & environmental sciences

For a reference copy of the document with all sections, see nature.com/documents/nr-reporting-summary-flat.pdf

Life sciences study design

All studies must disclose on these points even when the disclosure is negative.

Sample size	Three monkeys received MHCII-deficient RPE transplantation, another three monkeys received gene-edited MHCII-KO RPE transplantation, and one monkey received wildtype RPE transplantation.
Data exclusions	No data were excluded.
Replication	MHCII-deficient RPE transplantation was performed on three independent biological replicates; MHCII-KO RPE transplantation was performed on three independent biological replicates.
Randomization	Healthy monkeys were randomely assigned for each transplantation.
Blinding	Performers of the transplantation were also involved in data collection, so blinding was not possible.

Reporting for specific materials, systems and methods

We require information from authors about some types of materials, experimental systems and methods used in many studies. Here, indicate whether each material, system or method listed is relevant to your study. If you are not sure if a list item applies to your research, read the appropriate section before selecting a response.

Materials & experimental systems

n/a	Involved in the study	
☐	☒	Antibodies
☒	☐	Eukaryotic cell lines
☒	☐	Palaeontology and archaeology
☐	☒	Animals and other organisms
☒	☐	Human research participants
☒	☐	Clinical data
☒	☐	Dual use research of concern

Methods

n/a	Involved in the study	
☒	☐	ChIP-seq
☐	☒	Flow cytometry
☒	☐	MRI-based neuroimaging

Antibodies

Antibodies used

Anti-human HLA-class I, FITC Mouse 10 µl/test F5662 Sigma-Aldrich
Anti-human HLA-class II, FITC Mouse 10 µl/test MCA2497F AbD Serotec
Anti-human CD40, PE Mouse 5 µl/test 313006 BioLegend
Anti-human CD80 (B7-1), PE Mouse 5 µl/test 11-0809 eBioscience
Anti-human CD86 (B7-2), Alexa Fluor 488 Mouse 5 µl/test 53-0869 eBioscience
Anti-bestrophin Mouse 10 µl/test MAB5466 Millipore
Anti-MerTK rabbit 10 µg/test sc-67280 Santa Cruz
Anti-MiTF Mouse 10 µg/test ab80651 Abcam
Anti-Oct3/4 Mouse x200 sc-5279 Santa Cruz
Anti-Pax6 rabbit x200 PRB-278P Covance
Anti-PEDF Mouse 10 µg/test ab115489 Abcam
Anti-RPE65 Mouse 10 µg/test MAB5428 Millipore
Anti-tyrosinase Mouse x200 ab738 Abcam
Anti-ZO-1 rabbit x100 61-7300 Thermo Fisher
Mouse IgG1, isotype control - 1 µg/test ab170190 Abcam
Mouse IgG1, isotype control, PE - 5 µl/test 400112 BioLegend
Mouse IgG2a, isotype control, FITC - 2 µl/test 400210 BioLegend
Mouse IgG2b, isotype control, APC - 5 µl/test 400322 BioLegend
Rabbit IgG, isotype control - x200 02-6102 Thermo Fisher
Anti-human CD4, APC Mouse 5 µl/test 317416 BioLegend
Anti-human CD8a, APC Mouse 5 µl/test 17-0088 eBioscience
Anti-human CD11b, APC Rat 5 µl/test 13-091-241 Miltenyi Biotec
Anti-human CD20, APC Mouse 5 µl/test 130-097-619 Miltenyi Biotec

	Anti-human Ki-67, PE Mouse 5 µl/test 350504 BioLegend<br>Anti-CD3 Rabbit x100 ab16669 Abcam<br>Anti-Iba1 Rabbit x1000 019-19741 Wako<br>Anti-CD4 Rabbit X100 Ab133616 Abcam<br>Anti-MHC class II (HLA-DP, DQ, DR) Mouse x100 Nr.M0775 DakoCytomation<br>Alexa Fluor 488 anti-rabbit IgG (H+L) Goat x1000 A11034 tab<br>Alexa Fluor 546 anti-rabbit IgG (H+L) Goat x1000 A11035 Invitrogen<br>Alexa Fluor 647 anti-rabbit IgG (H+L) Goat x1000 A21245 Invitrogen<br>Alexa Fluor 546 anti-mouse IgG (H+L) Goat x1000 A11030 Invitrogen<br>anti-CIITA rabbit antibody (GENETEX, GTX129022 1:100),<br>anti-MHC class II mouse antibody (abeomics, 36-1236 1:500)<br>anti-αTubulin mouse antibody (SIGMA-ALDRICH, DM1A, 1:5000)
Validation	Reactivities against monkey cells were stated in manufacturers' datasheet or were validated in our preliminary studies.

Animals and other organisms

Policy information about studies involving animals; ARRIVE guidelines recommended for reporting animal research

Laboratory animals	cynomolgus macaques; Macaca fascicularis; male
Wild animals	The study did not involve wild animals.
Field-collected samples	The study did not involve samples collected from the field.
Ethics oversight	ARVO Statement for the Use of Animals in Ophthalmic and Vision Research; Guidelines of the RIKEN Animal Experiment Committee.

Note that full information on the approval of the study protocol must also be provided in the manuscript.

Flow Cytometry

Plots

Confirm that:

☒ The axis labels state the marker and fluorochrome used (e.g. CD4-FITC).

☒ The axis scales are clearly visible. Include numbers along axes only for bottom left plot of group (a 'group' is an analysis of identical markers).

☒ All plots are contour plots with outliers or pseudocolor plots.

☒ A numerical value for number of cells or percentage (with statistics) is provided.

Methodology

Sample preparation	Cells were peripheral blood mononuclear cells isolated from monkey blood samples. For Ki-67 staining, cells were fixed with 70% ethanol.
Instrument	BD Canto II Flow Cytometer
Software	BD FACSDiva software v6.0; FlowJo v10
Cell population abundance	The population was determined by staining with lymphocyte markers CD4, CD8, CD11b and NKG2A, and analyzing 1 million cells for each sample, which showed sufficient purity and abundance.
Gating strategy	The FSC/SSC gates were determined by our preliminary study to include the population positive for RPE markers (e.g. RPE65) or lymphocyte markers (e.g. CD4, CD8, CD11b or NKG2A). For Ki-67 staining, the gate was extended to higher FSC/SSC to include proliferating cells.

☒ Tick this box to confirm that a figure exemplifying the gating strategy is provided in the Supplementary Information.
